# Comprehensive Analysis of Gut Microbiota and Fecal Bile Acid Profiles in Children With Biliary Atresia

**DOI:** 10.3389/fcimb.2022.914247

**Published:** 2022-06-17

**Authors:** Ting Yang, Shen Yang, Jiawei Zhao, Peize Wang, Siqi Li, Yuyan Jin, Zhaozhou Liu, Xinyue Zhang, Yanan Zhang, Yong Zhao, Junmin Liao, Shuangshuang Li, Kaiyun Hua, Yichao Gu, Dingding Wang, Jinshi Huang

**Affiliations:** Department of Neonatal Surgery, Beijing Children’s Hospital, Capital Medical University, National Center for Children’s Health, Beijing, China

**Keywords:** biliary atresia, gut microbiome, bile acid spectrum, cholangitis, jaundice clearance

## Abstract

**Background:**

Biliary atresia (BA) is the most common cholestatic liver disease in neonates. Herein, we aimed at characterizing the gut microbiota and fecal bile acid profiles of BA patients, defining the correlations between them, and evaluating the relationship between the clinical pathogenesis and changes in the gut microbiota and bile acid profiles.

**Methods:**

A total of 84 fecal samples from BA patients (n = 46) and matched healthy controls (HCs, n = 38) were subjected to sequencing by 16S rRNA gene amplification, and fecal bile acid were analyzed by targeted metabolomics.

**Findings:**

Compared with the controls, a structural separation of the intestinal flora of BA patients was uncovered, which was accompanied by changes in the composition of fecal bile acids. In the BA group, *Actinobacillus*, *Monoglobus*, and *Agathobacter* were enriched in patients without cholangitis (*p* < 0.05). *Selenomonadaceae* and *Megamonas* were more abundant in patients without recurrent cholangitis episodes (*p* < 0.05), while *Lachnospiraceae* and *Ruminococcaceae* were enriched in patients with multiple recurrences of cholangitis (*p* < 0.05). Postoperative jaundice clearance was associated with *Campylobacter* and *Rikenellaceae* (*p* < 0.05), and tauroursodeoxycholic acid was associated with jaundice clearance (*p* < 0.001).

**Conclusion:**

BA patients are characterized by different compositions of gut microbiota and bile acids, and their interaction is involved in the process of liver damage in BA, which may be closely related to the occurrence of postoperative cholangitis and jaundice clearance.

## Introduction

Biliary atresia (BA) is a severe neonatal disease of the hepatobiliary system, in which the intrahepatic and extrahepatic bile ducts are occluded, resulting in cholestasis and progressive liver fibrosis (Kasahara et al., 2017; Lane and Murray, 2017). Even after successful Kasai surgery (portoenterostomy), the majority of children progress to end-stage cirrhosis (Asai et al., 2015). A study showed that the native liver survival rates at 10 and 20 years after Kasai was 70.7% and 61.5% respectively (Chung et al., 2021).

The enterohepatic axis is an anatomical and physiological bridge that connects the intestine and liver (Long et al., 2017). Intestinal microflora balance is the key to maintaining immune homeostasis of the gut-liver axis. Changes in intestinal permeability and gut bacteria profiles are related to many diseases, and dysbacteriosis is an important cause of liver diseases (Feenstra et al., 1985; Hiippala et al., 2018; Tripathi et al., 2018). Gut microbiota disorders have been found in a variety of liver diseases, such as alcoholic hepatitis, cirrhosis, ischemic liver injury, rejection in liver transplantation, and cholestatic liver diseases (Sun et al., 2017; Song et al., 2021; Song et al., 2021; Liu et al., 2021). Previous study has reported that disturbed gut microbiota structures can be observed during the early and late stages of BA (Song et al., 2021). Liver damage in BA is directly or indirectly exacerbated by the interplay between *Klebsiella*, *Veronella*, and *Enterococcus* enrichment and impaired tryptophan and bile acid metabolism (Song et al., 2021). Several studies have found the dysbiosis of the gut microbiome to be associated with BA, as well as an increase in the abundances of *Proteus*, *Enterococcus*, *Streptococcus*, and other opportunistic pathogens, while the abundances of some butyrate-producing bacteria were found to be reduced (Wang et al., 2019; Wang et al., 2020; Song et al., 2021).

Bile acids are produced by the liver from cholesterol through classical or alternative pathways, and primary bile acids are dehydroxylated by the gut microbiome to form secondary bile acids in the intestine. Individual bile acids have recently been found to be useful for the differentiation and assessment of liver injury (Fu et al., 2020). Plasma levels of selected bile acids were higher in BA patients than that in cholestatic non-BA patients; more specifically, plasma levels of taurochenodeoxycholic acid (TCDCA) and chenodeoxycholic acid (CDCA) were increased and decreased, respectively. Fecal bile acid levels were significantly lower in BA patients due to the disruption of normal biliary drainage, with a significant difference between BA and healthy control (HC) groups (Golden et al., 2018).

Previous studies have provided a preliminary description of the compositions of the gut microbiota and bile acids in patients with BA, but whether a correlation exists between the two is still unclear. In particular, there is a lack of knowledge on their association with important disease features of BA, such as post-Kasai cholangitis, jaundice clearance, and prognosis. Thus, a comprehensive analysis of the gut microbiota in a large BA cohort and a thorough exploration of its relationship with the bile acid profile are needed. Herein, we aimed at characterizing the gut microbiota and fecal bile acid profiles of BA patients compared with those of HCs, unraveling possible correlations between them and evaluating the relationship between the clinical pathogenesis of BA and changes in gut microbiota and bile acid profiles.

## Methods

### Study Group

Patients with BA (n = 46) and age-matched healthy controls (HC, n = 38) were recruited from Beijing Children’s Hospital, Capital Medical University, from October 2019 to June 2021. Forty-six patients with BA had their diagnosis based on intraoperative cholangiography and liver biopsy. The patients were enrolled with the following criteria: age less than 6 months; no remaining gastrointestinal diseases; no history of antibiotic and probiotic use within 2 weeks prior to enrollment. HC consists of children in the same age group as BA with non-digestive diseases, e.g. children with polydactylism, precocious heart disease, inguinal hernia, etc. Stool samples were freshly collected as soon as possible after admission preoperatively, and frozen them at -80°C within one hour. Follow-up and long-term sequelae were collected from outpatient and inpatient records, as well as *via* clinical and telephone communication. All patients with BA received antibiotics, ursodeoxycholic acid, steroids and probiotics post-operatively. This study was approved by the Ethics Committee of Beijing Children’s Hospital (2019-k-386), and informed consent was obtained from the guardians of each participant before sample collection.

### Gene Amplicon Sequencing of 16S rRNAs and Data Processing

DNA was extracted using the E.Z.N.A.^®^ soil DNA Kit (Omega Bio-tek, Norcross, GA, USA) according to the manufacturer’s instructions. The hypervariable V3-V4 regions of the bacterial 16S rRNA gene were amplified with 338F/806R primers using an ABI GeneAmp^®^ 9700 PCR thermocycler (ABI, CA, USA). Purified amplicons were pooled in equimolar amounts and paired-end sequenced on an Illumina MiSeq PE300 platform (Illumina, San Diego, USA) according to the standard protocols employed by Majorbio Bio-Pharm Technology Co. Ltd. (Shanghai, China). Raw 16S rRNA gene sequencing reads were demultiplexed, quality-filtered using fastp version 0.20.0 (Chen et al., 2018), and merged using FLASH version 1.2.7 (Magoč and Salzberg, 2011). Operational taxonomic units (OTUs) with a 97% similarity cutoff were clustered using UPARSE version 7.1 (Edgar, 2013), and chimeric sequences were identified and removed. Taxonomic classification of the sequences of each sample was carried out individually using RDP Classifier version 2.2 (Wang et al., 2007) according to the RDP database.

### Quantitative Analysis of Bile Acids

The bile acids from the human fecal samples were quantitatively conducted by Metabo-Profile Inc. (Shanghai, China) according to their previously published methods (Xie et al., 2015; Lan et al., 2016; Zheng et al., 2017). All of the bile acid standards were purchased from Steraloids Inc. (Newport, RI, USA) and TRC Chemicals (Toronto, ON, Canada) or synthesized from the MPB laboratory, and 6 stable isotope-labeled standards were obtained from C/D/N Isotopes Inc. (Quebec, Canada) and Steraloids Inc. (Newport, RI, USA). Ultra-performance liquid chromatography-tandem mass spectrometry (UPLC-MS/MS, ACQUITY UPLC-Xevo TQ-S, Waters Corp., Milford, MA, USA) was used to quantify for the bile acids in human fecal samples. Fecal samples (10 mg) were accurately weighed and homogenized with 20 µL of deionized water and 180 µL of acetonitrile/methanol (v/v=8:2) containing 6 internal standards for 3 min (BB24, Next Advance, Inc, Averill Park, NY, USA). After centrifugation at 13,500 rpm for 20 min at 4°C (Microfuge 20R, Beckman Coulter, Inc., Indianapolis, IN, USA). The supernatant (20 µL) was transferred to a 96-well plate and lyophilized in a freeze dryer (Labconco, Kansas City, MO, USA), and then diluted with 1:1 solvent mixture of acetonitrile/methanol (80/20) and ultrapure water, mixed, and centrifuged. 5 µL aliquot of the supernatant of each sample was used for UPLC-MS/MS analysis. The raw data files generated by UPLC-MS/MS analysis were processed using MassLynx software (v4.1, Waters, Milford, MA, USA), upon which they were integrated, standard curves were created, and all bile acids were quantified. The bile acids detected in this study as follows: 3-DHCA: 3-dehydrocholic acid; 6-ketoLCA: 6-ketolithocholic acid; 7-ketoDCA: 7-ketodeoxycholic acid; 12-ketoLCA: 12-ketolithocholic acid; βUDCA: 3β-ursodeoxycholic acid; C4: 7a-Hydroxy-cholestene-3-one; CA: cholic acid; CDCA: chenodeoxycholic acid; CDCA-3Gln: chenodeoxycholic acid-3-β-D-glucuronide; DCA: deoxycholic acid; GCA: glycocholic acid; GCDCA: glycochenodeoxycholate; GDCA: glycodeoxycholic acid; GHCA: glycohyocholate; GLCA: glycolithocholate; GUDCA: glycoursodeoxycholic acid; HCA: γ-muricholic acid/hyocholic acid; LCA: lithocholic acid; LCA-3S: lithocholic acid 3 sulfate; NorCA: Nor Cholic acid; TCA: taurocholic acid; TCDCA: taurochenodeoxycholate; TDCA: taurodeoxycholate; TUDCA: tauroursodeoxycholic acid; UDCA: ursodeoxycholic acid.

### Statistical Analysis

In this study, SPSS Statistics 26 software was used for statistical analysis of the data. Wilcoxon rank-sum test and Fisher’s exact test were used to identify statistical differences in continuity variables and categorical variables, respectively, between the two groups. *p* < 0.05 was defined as statistically significant. Redundancy analysis (RDA) was conducted to determine the effect of environmental factors on the structure of the intestinal flora. Principal coordinates analysis (PCoA) between species was used to evaluate the β diversity of the gut microbial composition. Linear discriminant analysis effect size (LEfSe) analysis using linear discriminant analysis (LDA) was used to determine the taxonomic differences between BA and HC groups. The community heatmap is a color gradient illustrating community composition and abundance information at the genus level. Unidimensional statistical analysis was used to screen for differential bile acids between BA and HC groups. The non-parametric Wilcoxon test was performed to analyze statistically significant differences at different taxonomic levels between the different cohorts. Spearman correlation coefficients were calculated to investigate the relationship between clinical parameters, microbial composition, and bile acids.

## Results

### Characteristics of Gut Microbiota Profiles in the Study Population

**Figure 1:**
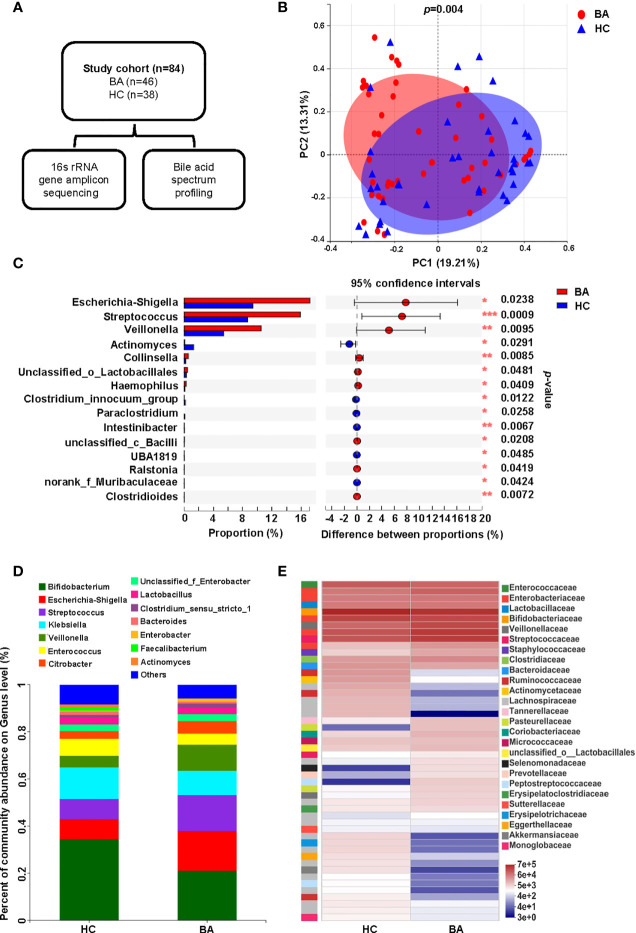
Fecal microbiome variations and composition in BA and HC groups. **(A)** Overview of the study design. **(B)** Beta diversity (principal coordinates analysis based on the Bray-Curtis distance of genus abundance) between the two groups. **(C)** Microbiota with significantly different abundances between the two groups at the genus level (*p < 0.05, **p < 0.01, and ***p < 0.001). **(D)** Bar diagram of the fecal microbial composition in the two groups. **(E)** Community heatmap presenting information on the genus-level composition and abundance of the community between the two groups, with color changes reflecting the similarities and differences in community composition. BA, biliary atresia; HC, health control; PCoA, principal coordinated analysis.

In this study, fecal samples from 84 individuals were sequenced after 16S rRNA gene amplification to assess the composition of the gut microbiome, and a targeted metabolomics approach was used to detect the bile acid composition in each fecal sample (**Figure 1A**). Common demographic data and clinical characteristics are shown in **Table 1**. A further RDA analysis was conducted to determine the effect of age, sex, mode of delivery, gestational age and feeding pattern on the structure of the intestinal flora (**Supplementary Figure 1**). The permutation test (**Supplementary Table 1**) showed that none of the above environmental factors had a significant effect (all *p* > 0.05) on the composition of the fecal microbiota, so we did not consider these confounding effects in the subsequent analysis. To investigate the differences in gut microbiota between patients with BA and HC individuals, we determined the changes in the gut microbiota of the two groups and evaluated the differences in community composition using PCoA based on Bray-Curtis differences, which showed that the overall microbial composition in the feces of the BA group deviated from that of the HC group (**Figure 1B**). LDA scores of the gut microbiota are shown in **Supplementary Figure 2A**, and the correlation between fecal microbiota composition and samples is shown in **Supplementary Figures 2B, C**. At the genus level, the BA and HC groups were enriched with 77 and 45 differential species, respectively. A specific list of differential species is shown in **Supplementary Table 2**. **Figures 1C**, **1D** show the composition of the fecal microbiota in BA and HC groups, indicating significant differences between the two groups at the genus level. Among them, *Bifidobacterium*, *Escherichia-Shigella*, *Klebsiella*, *Streptococcus*, and *Veillonella* were the dominant species in both the BA and HC groups. *Escherichia-Shigella*, *Streptococccus*, *Veillonella*, and *Citrobacter* were more abundant in the individuals of the BA group than in HC individuals, whereas *Bifidobacterium*, *Klebsiella*, and *Enterobacter* were more abundant in the case of the latter (**Figure 1D**). As shown in **Figure 1C**, *Escherichia-Shigella*, *Streptococccus*, and *Veillonella* were significantly enriched in the BA group, whereas *Actinomyces* was significantly less abundant in BA patients. These differences were determined to be statistically significant (all *p* < 0.05). **Figure 1E** shows the differences in microbial abundance between BA and HC groups, with *Faecalibacterium*, *Actinomyces*, *Agathobacter*, *Blautia*, *Eggerthella*, as well as other genera being of lower abundance in the BA group than in the HC group. In conclusion, the overall structure of the gut microbiota of BA patients was significantly different from that of the HC group.

**Table 1:** Clinical characteristics of the BA and HC patients.

Clinical characteristics	BA (n = 46)	HC (n = 38)	*p*-value
Female, n (%)	22 (47.8)	24 (63.2)	0.273
Age, days, median (IQR, Q1-Q3)	55.0 (40.8-75.0)	58.0 (46.5-115.5)	0.100
AST, U/L, median (IQR, Q1-Q3)	242.5 (137.4-338.0)	43.2 (37.1-67.9)	< 0.001
ALT, U/L, median (IQR, Q1-Q3)	131.0 (75.0-237.3)	24.6 (19.2-41.1)	< 0.001
GGT, U/L, median (IQR, Q1-Q3)	336.5 (185.3-723.1)	35.9 (18.1-103.6)	0.001
TBIL, μmol/L, median (IQR, Q1-Q3)	168.1 (131.0-202.2)	22.3 (9.9-71.0)	< 0.001
DBIL, μmol/L, median (IQR, Q1-Q3)	114.5 (83.1-146.1)	4.1 (1.9-13.8)	< 0.001
IBIL, μmol/L, median (IQR, Q1-Q3)	49.6 (42.3-67.2)	17.8 (7.8-34.1)	< 0.001
TBA, μmol/L, median (IQR, Q1-Q3)	114.3 (90.8-150.3)	7.1 (3.5-11.9)	< 0.001

ALT, alanine aminotransferase; AST, aspartate aminotransferase; BA, biliary atresia; DBIL, direct bilirubin; GGT, gamma-glutaryl transferase; HC, health control; IBIL, indirect bilirubin; TBA, total bile acid; TBIL, total bilirubin.

### Characteristics of Bile Acid Profiles in the Study Population

**Figure 2:**
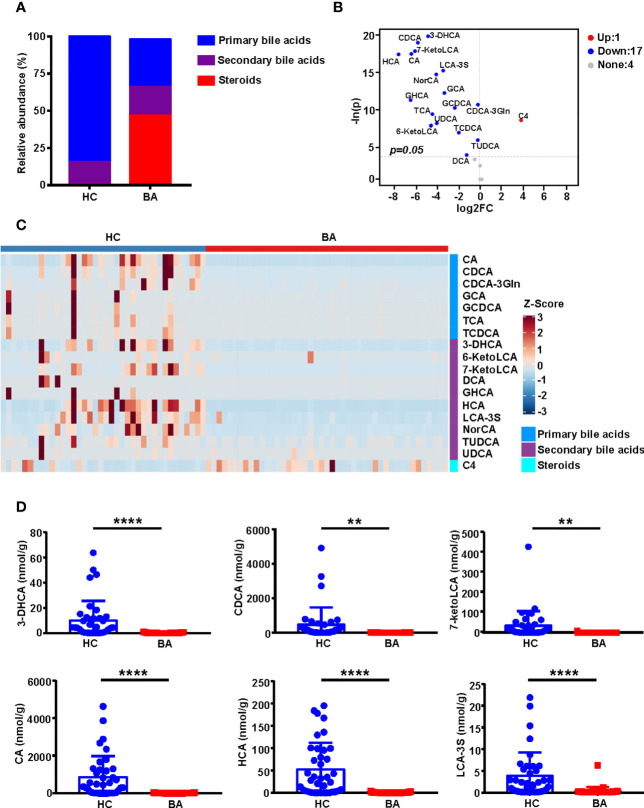
Bile acid profile composition in BA and HC groups. **(A)** Stacked histograms of the relative abundances of median values of various bile acids in the two groups of samples. **(B)** Volcano plot showing the differential metabolites screened based on a unidimensional statistical analysis. The thresholds in the plot were set as follows: *p* < 0.05 and between-group variation multiplier, absolute value of log_2_ fold change (FC) ≥ 0. A total of 18 differential metabolites were obtained from the unidimensional analysis according to the screening criteria. Bile acids elevated or decreased are highlighted in red and blue, respectively. **(C)** The heatmap highlights the difference in abundance of bile acid composition between the two groups, with the changes in color reflecting the similarities and differences between the different groups. **(D)** Box plots of the top 6 differential metabolites for unidimensional statistical analysis of *p* values (**p < 0.01 and ****p < 0.0001). 3-DHCA, 3-dehydrocholic acid; 6-ketoLCA, 6-ketolithocholic acid; 7-ketoLCA, 7-ketolithocholic acid; BA, biliary atresia; C4, 7a-Hydroxy-cholestene-3-one; CA, cholic acid; CDCA, chenodeoxycholic acid; CDCA-3Gln, chenodeoxycholic acid-3-β-D-glucuronide; DCA, deoxycholic acid; FC, Fold Change; GCA, glycocholic acid; GCDCA, glycochenodeoxycholate; GHCA, glycohyocholate; HC, health control; HCA, γ-muricholic acid/hyocholic acid; LCA-3S, lithocholic acid 3 sulfate; NorCA, nor cholic acid; TCA, taurocholic acid; TCDCA, taurochenodeoxycholate; TUDCA, tauroursodeoxycholic acid; UDCA, ursodeoxycholic acid.

**Figures 2A** and **2C** show statistically significant differences in the composition of primary bile acids, secondary bile acids, and steroids between BA and HC groups. The specific bile acid composition of each sample is shown in **Supplementary Figure 3A**. Compared to the HC group, the BA group was characterized by a lower abundance of primary bile acids and comparable levels of secondary bile acids but a higher abundance of steroids (**Figure 2A**). A total of 18 differential metabolites were screened based on unidimensional statistical analysis (all *p* < 0.05, **Figure 2C**). As shown in the volcano plot presented in **Figure 2B**, compared to the HC group, the metabolite C4, highlighted in the upper right corner, was elevated in BA patients, whereas 17 bile acids, including CDCA, CA, HCA, GCA, and TCA, which are presented in the upper left corner, were low in the BA group. Box plots of the top 6 differential metabolites ranked by unidimensional statistical analysis are shown in **Figure 2D**.

### Association Between Disease-Related Fecal Microbiota, Fecal Bile Acid Profiles, and Preoperative Serum Biochemical Indices

**Figure 3:**
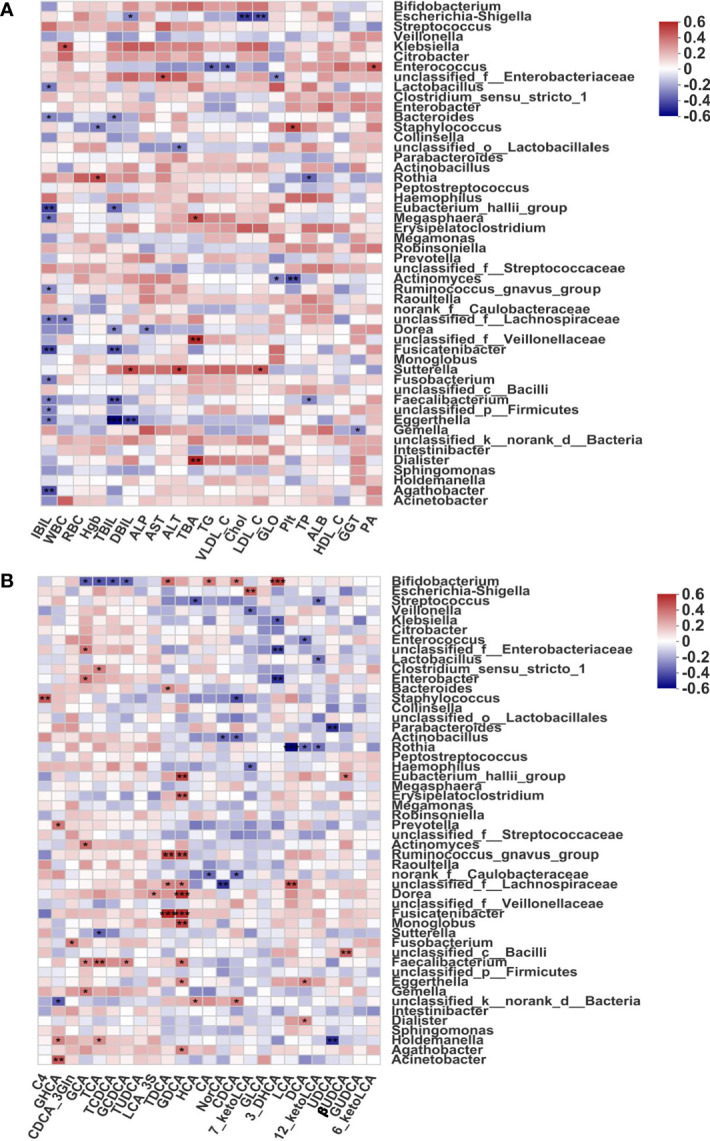
Associations of disease-linked microbiota with clinical indicators and bile acids. **(A)** Heatmap of Spearman correlation coefficients between the fecal microbiome and indicators of liver function. **(B)** Heatmap of Spearman correlation coefficients between the fecal microbiome and bile acid profiles in BA. Blue and red denote negative and positive correlations, respectively. The depth of the color indicates the correlation between the genus and environmental factors. *p < 0.05, **p < 0.01, ***p < 0.001. ALB, albumin; ALP, alkaline phosphatase; ALT, alanine aminotransferase; AST, aspartate aminotransferase; Chol, cholesterol; DBIL, direct bilirubin; GGT, gamma-glutaryl transferase; GLO, globulin; HDL_C, high-density lipoprotein cholesterol; Hgb, hemoglobin; IBIL, indirect bilirubin; LDL_C, low-density lipoprotein cholesterol; PA, prealbumin; Plt, platelet; RBC, red blood cell; TBA, total bile acid; TBIL, total bilirubin; TG, triglyceride; TP, total protein; VLDL_C, very low-density lipoprotein cholesterol; WBC, white blood cell; 3-DHCA, 3-dehydrocholic acid; 6-ketoLCA, 6-ketolithocholic acid; 7-ketoLCA, 7-ketolithocholic acid; 12-ketoLCA, 12-ketolithocholic acid; βUDCA, 3β-ursodeoxycholic acid; C4, 7a-hydroxy-cholestene-3-one; CA, cholic acid; CDCA, chenodeoxycholic acid; CDCA-3Gln, chenodeoxycholic acid-3-β-D-glucuronide; DCA, deoxycholic acid; GCA, glycocholic acid; GCDCA, glycochenodeoxycholate; GDCA, glycodeoxycholic acid; GHCA, glycohyocholate; GLCA, glycolithocholate; GUDCA, glycoursodeoxycholic acid; HCA, γ-muricholic acid/hyocholic acid; LCA, lithocholic acid; LCA-3S, lithocholic acid 3 sulfate; NorCA, nor cholic acid; TCA, taurocholic acid; TCDCA, taurochenodeoxycholate; TDCA, taurodeoxycholate; TUDCA, tauroursodeoxycholic acid; UDCA, ursodeoxycholic acid.

To evaluate the correlations between indicators of liver function and fecal microbiome profiles in BA, we calculated Spearman correlation coefficients (**Figure 3A**). Some intestinal microbiota genera were highly correlated with clinical indicators. More specifically, *Eggerthella, Eubacterium_halli_group, Fusicatenibacter, Bacteriodes*, and *Faecalibacterium* were found to be significantly negatively correlated with indicators of liver function (all *p* < 0.05); therefore, the above-mentioned bacteria with reduced abundance in BA patients were considered potentially beneficial genera. In addition, *Dialister*, *Megasphaera*, and an unclassified *Villonellaceae* strain showed significant positive correlations with total bile acids (TBA, *p* < 0.05), and these species might be potentially pathogenic. Furthermore, most of the fecal bile acids were negatively correlated with indicators of liver function (**Supplementary Figure 3B**).

In addition, one of the typical characteristics of BA is a decrease in fecal bile acids (Wang et al., 2020), which are synthesized in the liver from cholesterol, converted into primary bile acids, and metabolized in the intestine *via* the enterohepatic circulation. Normal biliary drainage was shown to be disrupted in BA, and we further found that the abundance of fecal bile acids was significantly decreased. Thus, we hypothesized that decreased biliary drainage could be one of the most important causes of gut microbiome dysbiosis among patients with BA. To investigate potential correlations between the gut microbiome and bile acids in BA, we calculated Spearman correlation coefficients and selected the 50 most abundant microorganisms for analysis. As shown in **Figure 3B**, *Eubacterium_halli_group*, *Erysipelatoclostridium*, *Ruminococcus_gnavus_group*, *Fusicatenibacter*, *Dorea*, and *Monoglobus* were all highly positively correlated with GDCA (all *p* < 0.01), a predictive marker for liver damage (Drzymała-Czyż et al., 2021). *Ruminococcus_gnavus_group* and *Fusicatenibacter* also showed a highly significant positive correlation with TDCA (all *p* < 0.01), *Bifidobacterium* showed a significant negative correlation with conjugated bile acids (GCA, TCA, TCDCA, and GCDCA; all *p* < 0.05), and *Rothia* was negatively correlated with secondary bile acids (DCA and LCA; both *p* < 0.05).

### Gut Microbiota Composition Correlates With Cholangitis After Kasai Surgery in BA Patients

**Figure 4:**
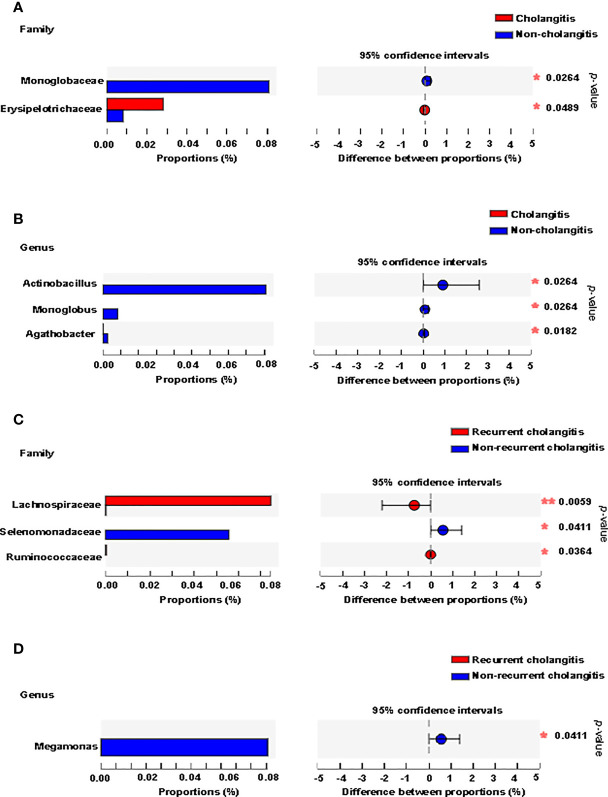
Fecal microbiota distribution of BA patients with cholangitis after Kasai surgery. Microbiota with significantly different abundances between the cholangitis and non-cholangitis groups at the family **(A)** and genus **(B)** levels are shown. Microbiota with significantly different abundances between recurrent cholangitis and non-recurrent cholangitis groups at the family **(C)** and genus **(D)** levels are presented. *p < 0.05, **p < 0.01.

Clinical signs (unexplained fever, recurrent jaundice, and acholic stools) and laboratory tests (raised bilirubin levels, elevated indicators of inflammation) are used to make the diagnosis of cholangitis. To further understand whether gut microbiota composition was associated with postoperative cholangitis after Kasai surgery among patients with BA, we divided the patients into two groups according to the occurrence of cholangitis. We analyzed the microbiota distribution at the family and genus levels and compared the differences between the groups. At the family level, *Erysipelotrichaceae* was enriched in the cholangitis group, whereas *Monoglobaceae* was enriched in the non-cholangitis group (**Figure 4A**). At the genus level, *Actinobacillus*, *Monoglobus*, and *Agathobacter* were enriched in the non-cholangitis group (**Figure 4B**).

Previous studies have suggested that the greater the number of recurrences of cholangitis, the more severe the patient’s liver damage (Chen et al., 2018). Thus, we divided patients with postoperative episodes of cholangitis into two groups based on cholangitis recurrence. Analysis of the gut community composition was also performed at the family and genus levels. At the family level, *Lachnospiraceae* and *Ruminococcaceae* were enriched in patients with multiple recurrences of postoperative cholangitis, while *Selenomonadaceae* was enriched in the group with non-recurrent cholangitis (**Figure 4C**). At the genus level, although *Megamonas* was characterized by an abundance score below 0.01 in both groups, the difference in relative abundance of *Megamonas* between the two groups was statistically significant (*p* = 0.041, **Figure 4D**).

### Gut Microbiota and Bile Acid Profiles Are Associated With Jaundice Clearance in BA

**Figure 5:**
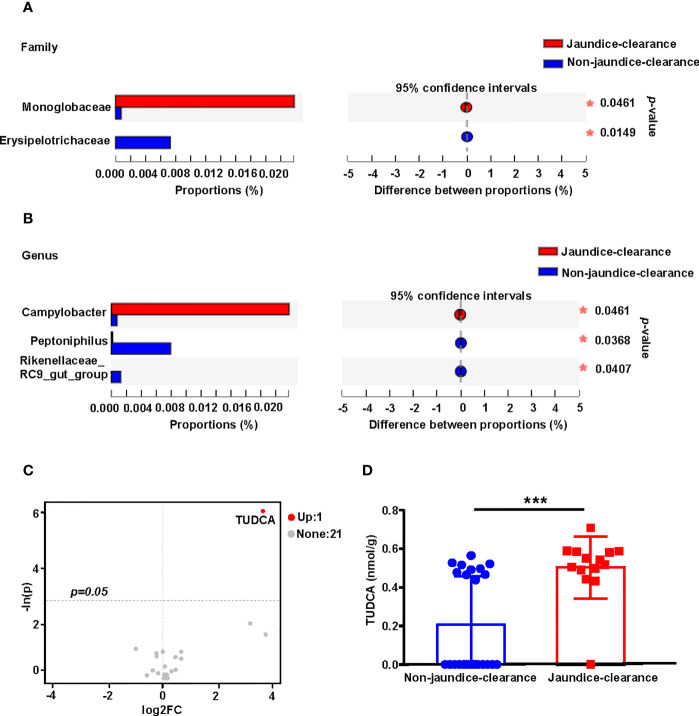
Fecal microbiota distribution and bile acid profile for patients with jaundice clearance at 6 months after Kasai surgery. Microbiota with significantly different abundances at the family **(A)** and genus **(B)** levels between jaundice-clearance and non-jaundice-clearance groups. **(C)** The volcano plot shows the differential metabolites screened based on a unidimensional statistical analysis. The thresholds in the plot were set as follows: *p* < 0.05 and between-group variation multiplier, absolute value of FC ≥ 0. **(D)** Box diagram of different levels of TUDCA between jaundice-clearance and non-jaundice-clearance groups. *p < 0.05, ***p < 0.001. FC, Fold Change; TUDCA, tauroursodeoxycholic acid.

Previous studies have confirmed that jaundice clearance after Kasai surgery is an important predictor of BA prognosis (Nakajima et al., 2018). In order to explore the relationship between the gut microbiota and bile acid profiles and jaundice clearance at 6 months after Kasai surgery (total bilirubin < 1.5 mg/dL), the BA patients were divided into jaundice-clearance and non-jaundice-clearance groups. The distribution of microbiota in the two groups was compared at the family and genus levels. At the family level, *Campylobacteraceae* was enriched in the jaundice-clearance group, while *Rikenellaceae* was enriched in the non-jaundice-clearance group (**Figure 5A**). At the genus level, *Campylobacter* was enriched in the jaundice-clearance group, whereas *Peptoniphilus* and *Rikenellaceae_RC9_gut_group* were enriched in the non-jaundice-clearance group (**Figure 5B**).

To understand the relationship between fecal bile acids and postoperative jaundice clearance in patients with BA, we screened for differential bile acids between the two groups based on unidimensional statistical analysis. As shown in the volcano plot in **Figure 5C**, only one differential bile acid was identified, with TUDCA highlighted in red in the upper right corner of the jaundice-clearance group compared to the non-jaundice-clearance group. **Figure 5D** also shows the increased level of TUDCA in the non-jaundice-clearance group.

## Discussion

In this study, we performed a comprehensive multi-omics analysis of the gut microbiota and bile acid compositions in BA. There is growing evidence in support of gut barrier dysfunction and dysbiosis constituting important causes of diseases affecting the liver as well as other organs (Tang et al., 2018; Wei et al., 2020). Bile acids, important substances associated with liver injury and cholestatic disease, are currently being investigated for their potential to serve as identifiers of cholestatic disease (Zhou et al., 2012). In this study, we explored the relationship between gut microbiota and bile acid composition in BA and its impact on BA pathogenesis.

Gut microbiota entering bile through the gut-liver axis or changes in the composition of the gut microbiota can lead to the activation of mucosal immune responses, which in turn leads to bacterial translocation and immune cell migration to the liver, thereby damaging the biliary tract and hepatocytes, consequently being associated with inflammation-mediated liver injury (Tajeddin et al., 2016; Yang et al., 2020). Compared to HC individuals, the fecal microbial compositions of BA patients were significantly distinct, suggesting that a disruption of the gut microbiota occurs in BA. In terms of specific genus composition, the abundances of *Escherichia-Shigella*, *Streptococccus*, and *Veillonella* were significantly increased in the BA group. *Escherichia-Shigella* has been shown to be increased in abundance in the feces of patients with ulcerative colitis (UC) (Kojima et al., 2012). *Streptococcus* and *Escherichia-Shigella* have been proven to be potential risk factors for UC deterioration. The severity of UC has been shown to be positively correlated with the abundance of *Escherichia-Shigella* in the inflamed mucosa (Kojima et al., 2012). Furthermore, increased abundance of *Escherichia-Shigella* has also been demonstrated in animal models of UC and was previously shown to be positively correlated with serum IL-6 levels (Jialing et al., 2020). indicating that *Escherichia-Shigella* is involved in the development of inflammatory diseases. *Streptococccus* is a major human pathogen that mainly causes localized infections of the skin and mucous membranes, which can adsorb high molecular weight kininogen in plasma, activate contact factors, and induce bradykinin release (Köhler et al., 2020). *Veillonella* is a bile acid-sensitive bacterium that is enriched when bile acids are inhibited. It has been reported in previous studies that bile acids are inhibited in the intestine of patients with cirrhosis and that *Veillonella* is enriched in the intestinal flora of patients with cirrhosis (Chen et al., 2016; Oh et al., 2020; Loomba et al., 2021; Song et al., 2021). Therefore, we suggest that the above-mentioned flora may be potentially pathogenic in BA. In contrast, *Actinomyces* is a normal bacterium colonizing the gastrointestinal tract, which cannot release exotoxins but produces formate, acetate, succinate, and lactate (Li et al., 2018). The metabolic potential of *Actinomyces* may be to break down and recycle organic matter in the human gastrointestinal system (Hanning and Diaz-Sanchez, 2015). In the present study, *Actinomyces* was significantly less abundant in BA patients; however, there is a lack of relevant studies on the biological functions of *Actinomyces* in the gastrointestinal system.

The intestinal microbiota is closely linked to the bile acid profile. Bile acids are synthesized in the liver and secreted into the small intestine as free or conjugated forms, where the microbiota deconjugates bile acids and converts primary bile acids into secondary bile acids (Camilleri and Gores, 2015). The depletion of bile acids in the gut and liver fibrosis may be major factors inducing dysbiosis of the intestinal flora. Zhou et al. found significant differences in various bile acids and TCDCA/CDCA ratios in serum samples from patients suffering from BA or neonatal hepatitis syndrome (Zhou et al., 2015). Another study showed that the abundance of bile acids were lower in the feces of patients with cholestasis than in that of HC individuals, with the greatest reduction in bile acid abundance being in BA patients (Zhou et al., 2015). Our data showed that the abundance of primary bile acids in feces was generally lower in patients with BA than in the HC individuals, whereas the abundance of steroids followed the reverse trend.

Patients with BA have clinically significant hyperbilirubinemia due to biliary occlusion, resulting in cholestasis and hepatic fibrosis. In our study, some intestinal genera were highly correlated with clinical indicators of liver disease. Specifically, *Eggerthella*, *Eubaterium_halli_group*, *Fusicatenibacter*, *Bacteriodes*, and *Faecalibacterium* were negatively correlated with indicators of liver function. Previous studies have shown that *Eggerthella* (Cho et al., 2016) and *Bacteriodes* (Wexler, 2007) play critical roles in liver metabolism and detoxification processes. *Faecalibacterium* is an important component of the gut microbiota and the most important butyric acid-producing bacterium in the human colon. Moreover, drugs and nutrients increase the levels of healthy flora in the human gut, including *Faecalibacterium (* Spanogiannopoulos et al., 2016 *).* A decrease in *Faecalibacterium* is currently thought to exacerbate inflammatory processes, and a negative association between *Faecalibacterium* and intestinal inflammation has been reported in some studies (Miquel et al., 2013). Therefore, we hypothesize that the above-mentioned genera exert potentially beneficial effects in BA. Conversely, *Dialister* and *Megasphaera* are both genera associated with inflammatory diseases, such as oral infections, pneumonia, and vaginitis (Rôças and Siqueira, 2006; Ravel et al., 2021). According to our data, *Dialister* and *Megasphaera* showed a significant positive association with TBA, thus representing potential pathogens in BA.

Cholangitis is the most common complication in patients with BA after Kasai surgery, with an incidence of 30%-70% (Gunadi et al., 2018). Several studies have suggested that cholangitis may be a risk factor for poor prognosis in patients with post-Kasai BA (Jiang et al., 2018). Cholangitis can lead to increased cholestasis and accelerated liver injury. An increasing number of studies have reported a potential role for *Erysipelotrichaceae* in human physiology and disease processes, such as enrichment after antibiotic treatment (Dinh et al., 2015). In this study, *Erysipelotrichaceae* was found to be significantly enriched in the feces of patients who had developed cholangitis after surgery. This indicated that a high abundance of *Erysipelotrichaceae* might increase the occurrence of cholangitis. *Monoglobus*, a pectin-degrading bacterium in the human colon (Kim et al., 2019), was enriched in non-cholangitis patients, along with *Actinobacillus*. Previous studies have suggested that the greater the number of cholangitis recurrences, the more severe will the patient’s liver damage be (Chen et al., 2018), The etiology of cholangitis is unknown, but based on the available studies, it is thought that intestinal bacterial migration and inflammatory responses may be dominant factors. In our study, *Lachnospiraceae* was observed to be enriched in the feces of patients with multiple postoperative recurrences of cholangitis. *Lachnospiraceae* is a major producer of short-chain fatty acids, but different taxa have also been associated with different intestinal and extraintestinal diseases, and the physiological effects on hosts are often inconsistent across studies (Vacca et al., 2020). In liver disease, enrichment of *Lachnospiraceae* may lead to mucosal immune dysregulation, promoting lymphocyte activation and increasing intestinal permeability. *Lachnospiraceae* was significantly increased in patients with primary sclerosing cholangitis-inflammatory bowel disease compared to HCs (Zhu et al., 2013), In addition, *Selenomonadaceae* was enriched in the intestines of patients with a single episode of cholangitis. *Megamonas* was not the dominant flora in patients with postoperative cholangitis after Kasai but was significantly enriched in the feces of patients with non-multiple, recurrent cholangitis. A higher abundance of *Megamonas* in control individuals has now been demonstrated by animal studies of intestinal inflammation (Maldonado-Contreras et al., 2020). Based on these findings, further research into *Megamonas* and its potentially beneficial effects are warranted in the future.

Jaundice-free survival is an important influencing factor for native liver survival after a Kasai procedure (Hukkinen et al., 2018; Pakarinen et al., 2018), which was defined as a total bilirubin (TBIL) < 1.5 mg/dL (Bezerra et al., 2014). *Rikenellaceae* abundance, previously reported to be significantly negatively correlated with fecal bile acids (Ikeda et al., 2020), was also confirmed in the present study; *Rikenellaceae* was found to be significantly enriched in the non-jaundice-clearance group. *Campylobacter* was enriched in the jaundice-clearance group, but the specific mechanism involved was not determined. The TUDCA level was higher in the jaundice-clearance group. Bear bile is thought to have anti-inflammatory, anti-bacterial, and gallstone-dissolving effects, and the bile acids in bear bile are in the form of taurine conjugates, of which the beneficial components are probably TUDCA and UDCA. Both have been shown to be potent cholestatic agents in animal studies (Cho et al., 2014; Li et al., 2016). TUDCA, the specific bile acid in bear bile, is a hydrophilic bile acid that is synthesized in the UDCA conjugation pathway and functions as a secretagogue and immunomodulator. The results of this study also support a beneficial effect of UDCA treatment after Kasai procedures in patients with BA.

There are several limitations to this study that have to be addressed. Firstly, this was a single-center study with a relatively small sample size, which may have influenced the results. Future multi-center studies are needed to validate the data presented. Secondly, the use of 16S rRNA amplification and sequencing in this study may have caused limitations with respect to functional prediction analysis. To compensate for this, we analyzed the fecal bile acid profiles. Thirdly, although our data showed an association between microbial communities, bile acid profiles, and clinical indicators, it did not yield a defined causal relationship. Further dissection of the potential mechanisms underlying the interaction is necessary. Finally, collect stool sample again after Kasai during the follow-up and looked at the bile acid and microbiome again was necessary in the future.

Our study describes alterations in the gut microbiome and bile acids in BA, and for the first time, we describe alterations in patients with postoperative cholangitis and jaundice clearance. In addition, we identified unique microbial-bile acid interactions in feces of BA patients, providing a possible disease-specific mechanism for future studies.

## Data Availability Statement

The datasets presented in this study can be found in online repositories. The names of the repository/repositories and accession number(s) can be found in the article/**Supplementary Material**.

## Ethics Statement

The studies involving human participants were reviewed and approved by the Ethics Committee of Beijing Children’s Hospital. Written informed consent to participate in this study was provided by the participants’ legal guardian/next of kin.

## Author Contributions

JSH, TY and SY conceptualized and supervised the study; TY, SY, YZ, JML, SSL, KYH, YCG and DDW managed the resources. JSH, TY and SY developed the methodology. TY, SY, JWZ, PZW, SQL, ZZL, YYJ, XYZ and YNZ performed the investigation. TY and SY wrote the manuscript. JSH reviewed and edited the manuscript. All authors contributed the article and approved the submitted version.

## Funding

This work was supported by the National Natural Science Foundation of China grants (#81660092).

## Conflict of Interest

The authors declare that the research was conducted in the absence of any commercial or financial relationships that could be construed as a potential conflict of interest.

## Publisher’s Note

All claims expressed in this article are solely those of the authors and do not necessarily represent those of their affiliated organizations, or those of the publisher, the editors and the reviewers. Any product that may be evaluated in this article, or claim that may be made by its manufacturer, is not guaranteed or endorsed by the publisher.

## Supplementary Material

Supplementary Figure 1 RDA analysis of the effect of environmental factors on the fecal microbiota structure. BA, biliary atresia; HC, health control; RDA, redundancy analysis.

Supplementary Figure 2 Histogram of LDA scores of the gut microbiota and the correlations between fecal microbiota structure and samples. **(A)** The composition of the gut microbiota between the HC and BA groups was analyzed by LEfSe. Histograms of the LDA scores calculated from the different abundances of microbes between the two groups. The critical point of the LDA score was 2.0, indicating a significant difference. Correlations between fecal microbiota structure and samples form BA **(B)** and HC **(C)** groups. BA, biliary atresia; HC, health control; LEfSe, linear discriminant analysis effect size; LDA, linear discriminant analysis.

Supplementary Figure 3 Bile acid composition of each sample and correlation of bile acids in feces with clinical indicators. **(A)** Stacked histograms of relative abundances of various bile acids in each sample. **(B)** Heatmap of the Spearman correlation coefficients between fecal bile acid compositions and indicators of liver function. ALP, alkaline phosphatase; ALT, alanine aminotransferase; AST, aspartate aminotransferase; BA, biliary atresia; Chol, cholesterol; DBIL, direct bilirubin; GGT, gamma-glutaryl transferase; HDL_C, high-density lipoprotein cholesterol; HC, health control; IBIL, indirect bilirubin; LDL_C, low-density lipoprotein cholesterol; PA, prealbumin; TBA, total bile acid; TBIL, total bilirubin; TG, triglyceride; TP, total protein; VLDL_C, very low-density lipoprotein cholesterol; 3-DHCA, 3-dehydrocholic acid; 6-ketoLCA, 6-ketolithocholic acid; 7-ketoLCA, 7-ketolithocholic acid; 12-ketoLCA, 12-ketolithocholic acid; βUDCA, 3β-ursodeoxycholic acid; C4, 7a-hydroxy-cholestene-3-one; CA, cholic acid; CDCA, chenodeoxycholic acid; CDCA-3Gln, chenodeoxycholic acid-3-β-D-glucuronide; DCA, deoxycholic acid; GCA, glycocholic acid; GCDCA, glycochenodeoxycholate; GHCA, glycohyocholate; GUDCA, glycoursodeoxycholic acid; HCA, γ-muricholic acid/hyocholic acid; LCA, lithocholic acid; LCA-3S, lithocholic acid 3 sulfate; NorCA, nor cholic acid; TCA, taurocholic acid; TCDCA, taurochenodeoxycholate; TUDCA, tauroursodeoxycholic acid; UDCA, ursodeoxycholic acid.
