# Supplementary figures and images for: Comprehensive Analysis of Gut Microbiota and Fecal Bile Acid Profiles in Children With Biliary Atresia
Source: Front Cell Infect Microbiol. 2022 Jun 17;12:914247. doi: 10.3389/fcimb.2022.914247 (PMC9247268; doi:10.3389/fcimb.2022.914247)

Supplementary Figure 1

A

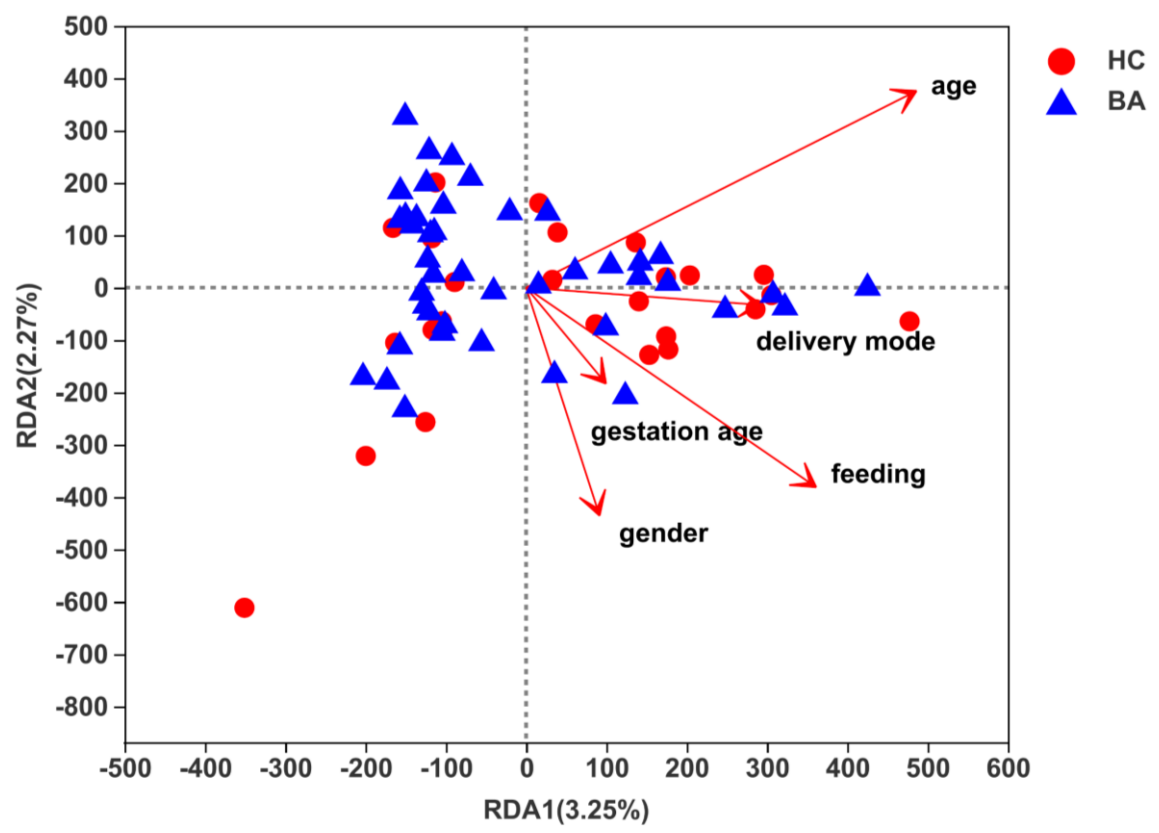

Supplementary Figure 2

A

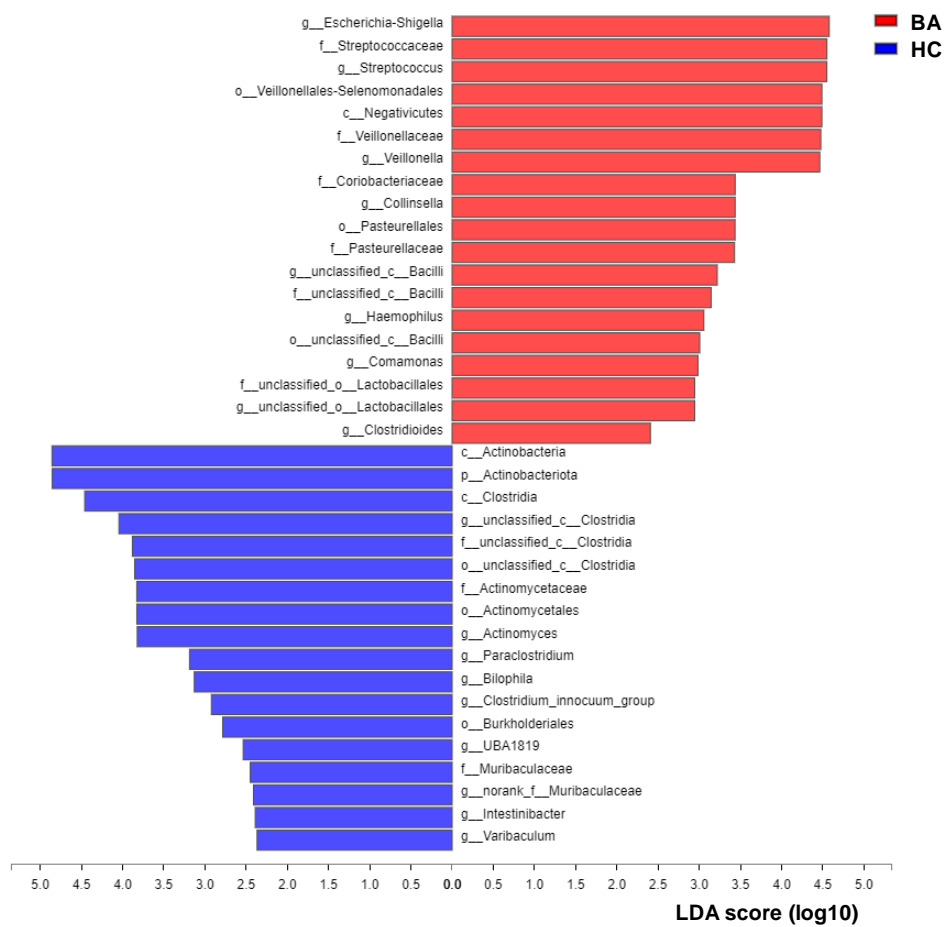

B

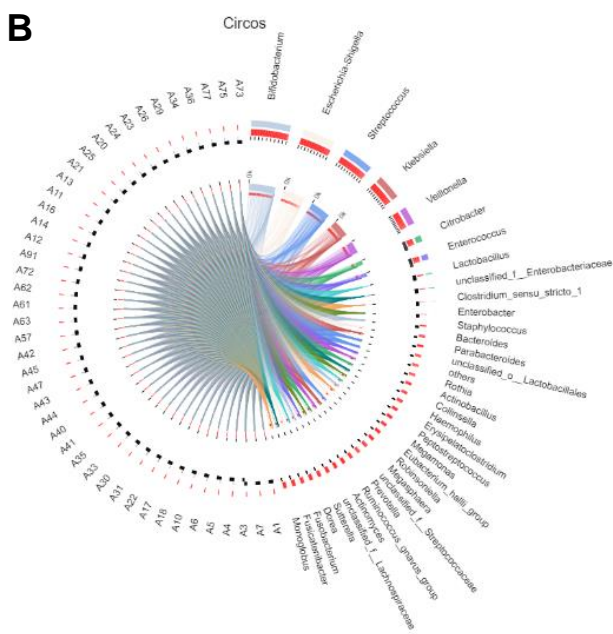

C

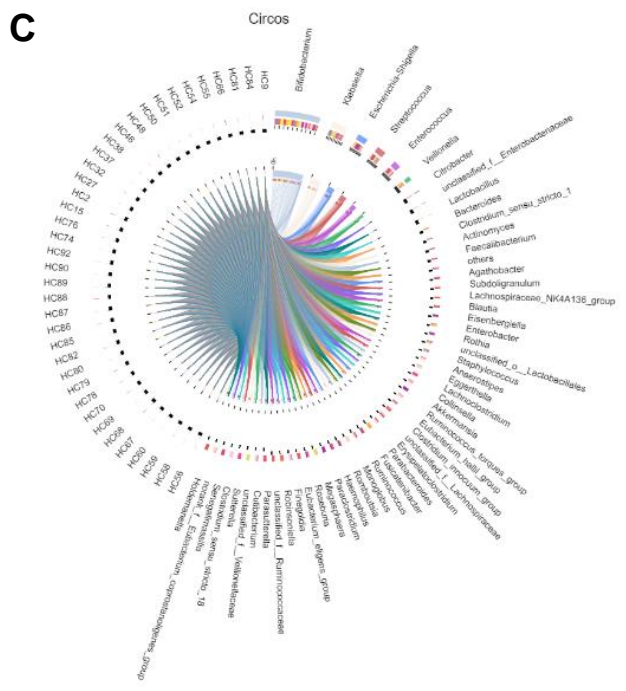

**A**

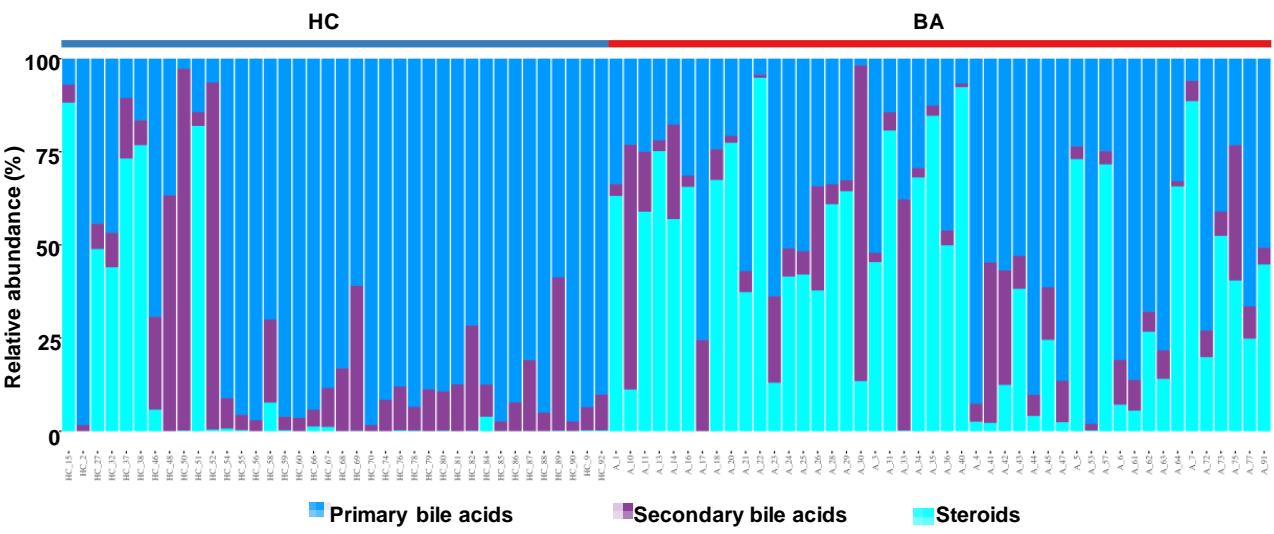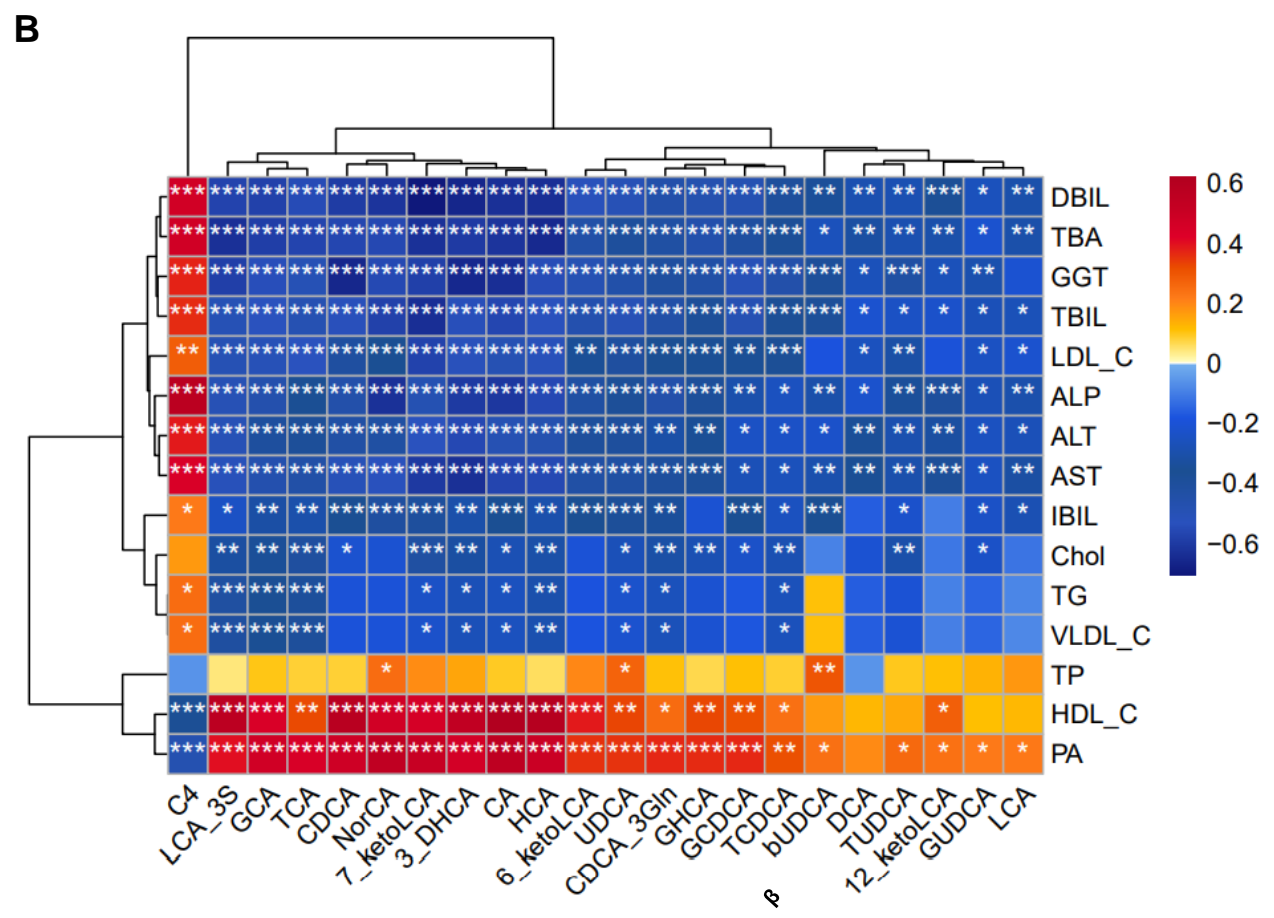

Supplement: Supplementary Figure 1 — RDA analysis of the effect of environmental factors on the fecal microbiota structure. BA, biliary atresia; HC, health control; RDA, redundancy analysis. [file Image_1.pdf]
