# Supplementary material for: Comprehensive Analysis of Gut Microbiota and Fecal Bile Acid Profiles in Children With Biliary Atresia
Source: Front Cell Infect Microbiol. 2022 Jun 17;12:914247. doi: 10.3389/fcimb.2022.914247 (PMC9247268; doi:10.3389/fcimb.2022.914247)
Supplement: Supplementary file 2 [file Table_1.docx]

**Supplementary Table 1. The permutation test of the effect of environmental factors on the composition of the fecal microbiota**

| **Environmental factors** | **RDA1** | **RDA2** | **r^2^** | ***p*-value** |
| --- | --- | --- | --- | --- |
| Feeding pattern | 0.5747 | -0.8184 | 0.0697 | 0.087 |
| Gender | 0.1721 | -0.9851 | 0.0546 | 0.158 |
| Delivery mode | 0.9836 | -0.1805 | 0.018 | 0.563 |
| Gestation age | 0.3829 | -0.9238 | 0.0108 | 0.713 |
| Age | 0.6905 | 0.7233 | 0.0873 | 0.056 |

RDA: redundancy analysis
