# Supplementary material for: Comprehensive Analysis of Gut Microbiota and Fecal Bile Acid Profiles in Children With Biliary Atresia
Source: Front Cell Infect Microbiol. 2022 Jun 17;12:914247. doi: 10.3389/fcimb.2022.914247 (PMC9247268; doi:10.3389/fcimb.2022.914247)
Supplement: Supplementary file 3 [file Table_2.docx]

**Supplementary Table 2. Differential genera in BA and HC groups**

| **HC** | **BA** |
| --- | --- |
| g_Anaerofilum | g_unclassified_f_Eggerthellaceae |
| g_Ezakiella | g_Tropheryma |
| g_Eubacterium_siraeum_group | g_Hydrogenoanaerobacterium |
| g_unclassified_c_Alphaproteobacteria | g_Brachybacterium |
| g_Clostridium_sensu_stricto_18 | g_norank_f_p-2534-18B5_gut_group |
| g_Propionimicrobium | g_Holdemania |
| g_Novosphingobium | g_Prevotellaceae_UCG-003 |
| g_Epulopiscium | g_Prevotellaceae_UCG-001 |
| g_Pedomicrobium | g_norank_f_UCG-010 |
| g_Actinotignum | g_Prevotellaceae_UCG-004 |
| g_Oscillospira | g_Enorma |
| g_Sphingoaurantiacus | g_Pedobacter |
| g_Moryella | g_Vulcaniibacterium |
| g_GCA-900066575 | g_unclassified_c_Actinobacteria |
| g_Bilophila | g_Anaerotruncus |
| g_MND1 | g_unclassified_o_Bacteroidales |
| g_Prevotellaceae_NK3B31_group | g_Catenibacterium |
| g_Sellimonas | g_Thermus |
| g_Mucispirillum | g_Eubacterium_ruminantium_group |
| g_Oribacterium | g_Dielma |
| g_Leptotrichia | g_Shuttleworthia |
| g_norank_f_Clostridium_methylpentosum_group | g_Lachnospiraceae_AC2044_group |
| g_Peptococcus | g_unclassified_p_Proteobacteria |
| g_Gemmobacter | g_Sharpea |
| g_norank_f_norank_o_Micavibrionales | g_Sphingopyxis |
| g_Anoxybacillus | g_UCG-009 |
| g_Hirschia | g_Deinococcus |
| g_Eubacterium | g_Aerococcus |
| g_Edaphobaculum | g_Dubosiella |
| g_unclassified_c_Clostridia | g_Phycicoccus |
| g_Scardovia | g_Lachnospiraceae_XPB1014_group |
| g_Oligoflexus | g_Parascardovia |
| g_norank_f_Gemmatimonadaceae | g_Allorhizobium-Neorhizobium-Pararhizobium-Rhizobium |
| g_Pseudoxanthomonas | g_Ahniella |
| g_Solobacterium | g_Arthrobacter |
| g_F0332 | g_Anaeroglobus |
| g_Paraclostridium | g_norank_f_norank_o_norank_c_KD4-96 |
| g_Dysgonomonas | g_Streptobacillus |
| g_CHKCI001 | g_Selenomonas |
| g_Coriobacteriaceae_UCG-002 | g_Bdellovibrio |
| g_Faecalitalea | g_Bergeyella |
| g_norank_f_Desulfovibrionaceae | g_Solirubrobacter |
| g_Christensenella | g_Sediminibacterium |
| g_Gaiella | g_Olsenella |
| g_Varibaculum | g_Tuzzerella |
|  | g_Tepidimonas |
|  | g_Micrococcus |
|  | g_Anaerofustis |
|  | g_Comamonas |
|  | g_Stomatobaculum |
|  | g_Luteimonas |
|  | g_norank_f_norank_o_Rhodospirillales |
|  | g_ASF356 |
|  | g_norank_f_F082 |
|  | g_Moraxella |
|  | g_TM7a |
|  | g_Tsukamurella |
|  | g_Rubrobacter |
|  | g_norank_f_JG30-KF-CM45 |
|  | g_unclassified_f_Planococcaceae |
|  | g_Acetitomaculum |
|  | g_Jeotgalicoccus |
|  | g_Alishewanella |
|  | g_unclassified_f_Oscillospiraceae |
|  | g_Cellulomonas |
|  | g_unclassified_f_Microbacteriaceae |
|  | g_Flavobacterium |
|  | g_Pyrinomonas |
|  | g_Brevibacterium |
|  | g_Pyramidobacter |
|  | g_Gardnerella |
|  | g_Sorangium |
|  | g_unclassified_f_Prevotellaceae |
|  | g_Treponema |
|  | g_Butyrivibrio |
|  | g_norank_f_norank_o_Clostridia_vadinBB60_group |
|  | g_Kocuria |

BA, biliary atresia; HC, health control.
